# Epigenetic Inflammation Supports Tumorigenesis: Activator Protein 1 Drives Stem Cell Memory to Augment Colitis and Cancer

**DOI:** 10.34133/research.1433

**Published:** 2026-09-30

**Authors:** Lingyi Wu, Jingjing Chen, Hu Zhang, Min Wu

**Affiliations:** ^1^ Department of Gastroenterology, West China Hospital, Sichuan University, Chengdu, China.; ^2^ Joint Research Centre on Medicine, The Second Affiliated Hospital of Wenzhou Medical University, and Wenzhou Institute, University of Chinese Academy of Sciences, Wenzhou, China.; ^3^ Tianfu Jincheng Laboratory, Chengdu, China.

## Abstract

A recent study published in *Nature* reveals that colonic stem cells retain long-term memory via activator protein 1 transcription-factor-mediated epigenetic remodeling following the resolution of inflammation. Chronic inflammation is an established risk factor for cancer; however, the mechanisms through which tissues maintain a memory of inflammatory history to drive malignant transformation after resolution remain largely elusive. This primed state markedly accelerates tumorigenesis upon the acquisition of oncogenic mutations. Chronic inflammation, exemplified by ulcerative colitis, is a key driver of colorectal cancer. Contrary to the classical view that inflammation promotes tumorigenesis via DNA damage, Nagaraja et al. showed that inflammation leaves genomic imprints on colonic stem cells, lowering their threshold for malignant transformation upon subsequent oncogenic stimuli. First discovered in the innate immune system, inflammatory memory has since been confirmed in epithelial barrier cells and stem cells. Research indicates that following inflammatory exposure, cells retain lasting epigenetic imprints through the remodeling of chromatin, which is the complex interaction and also binding product of DNA and proteins. The specific mechanisms may involve alterations in chromatin accessibility and the modification of histones that are proteins that bind to DNA. This discovery provides a feasible approach to investigating the mechanisms underlying the interplay between inflammation, epigenetic memory, and tumorigenesis‌, deepening our understanding of tumor progression.

Epigenetic memory of inflammation is defined as a stable, heritable, and cell-intrinsic chromatin remodeling state induced by a history of inflammatory exposure. This phenomenon involves persistent alterations in chromatin accessibility, transcription factor (TF) motif binding, and DNA methylation patterns that remain in stem cells long after the resolution of acute inflammation [1]. This persistent molecular state is specifically mediated by the activator protein 1 (AP-1) TF complex. Chronic inflammation driven by autoimmune diseases, persistent infections, smoking, and other factors markedly elevates cancer risk [2–7]. Patients with ulcerative colitis face a 2- to 5-fold higher risk of developing colorectal cancer than the general population, with particularly elevated risk among those who develop the disease in childhood [8,9]. Inflammation-induced epigenetic memory offers a plausible explanation for this clinical conundrum. Serving as a critical “bridge” between inflammation and tumors, epigenetic memory not only enables cells to maintain a tumor-susceptible state following the resolution of inflammation but also can be passed on to daughter cells through cell division, creating a long-term carcinogenic risk. This finding reshapes our understanding of the inflammation–cancer relationship [10,11].

Nagaraja et al. [1] simulated 3 disease stages representing the acute injury, chronic injury, and recovery phases of the intestine by administering 3 cycles of low-dose dextran sulfate sodium. Observations indicated that mice exhibited recovery in body weight, epithelial crypt architecture, and immune infiltration 21 d after the cessation of dextran sulfate sodium treatment. Joint analysis of chromatin accessibility and gene expression profiles in 52,540 single cells via the novel method SHARE-seq revealed that stem cells up-regulated interferon signaling and other immunomodulatory genes during the acute phase, whereas wound healing pathways were activated during the chronic phase. Colonic stem cells retained profound molecular imprints 102 d after the complete resolution of inflammation. Notably, while the expression of the vast majority of inflammation-related genes returned to baseline levels following recovery, the epigenome of these stem cells underwent enduring structural reorganization.

As single-cell sequencing, spatial transcriptomics, and epigenomics advance, integrated multi-omics has become a key strategy for unraveling cancer pathogenesis, enabling comprehensive dissection of the complex regulatory network linking inflammation, epigenetic memory, and cancer [12–14]. To establish the cell-intrinsic and heritable nature of this memory, Nagaraja et al. developed SHARE-TRACE to simultaneously profile clonal lineage, gene expression, differentiation state, and finally chromatin accessibility. A modified cellular barcoding approach was implemented based on single-cell sequencing technologies, enabling simultaneous analysis of the transcriptome and epigenome across over 50,000 cells and 172 clones. Building upon the logic that cells within the same clone exhibit higher similarity than randomly selected cells, the investigators employed permutation-based statistical methods to sensitively quantify the clonal heritability of epigenetic states during cell division. This analysis revealed the inheritance patterns of different TF motif families at the clonal level and confirmed that this intrinsic heritability operates independently of prior inflammatory exposure. Tracing experiments in colitis-derived organoids revealed that inflammatory memory persists through successive stem cell divisions without the involvement of immune cells. These findings strongly support that this memory originates from autonomous epigenetic encoding rather than lingering microenvironmental cues.

Having established the autonomous heritability of this memory, the investigators further searched for the key driving factors that mediate this process. Comparative analysis elucidated that although ETS and CTCF motifs exhibit memory, they lack clonal heritability and are likely driven by the extracellular environment. In contrast, AP-1 demonstrated substantial clonal memory and heterogeneity. This state remained stable across different clones within the same microenvironment, confirming its maintenance by cell-intrinsic mechanisms rather than regulation by paracrine signals. Mixed linear models further confirmed that clonal identity accounts for the majority of the variance in motif accessibility. These findings indicate that the variability between individual stem cells regarding specific motif families can be propagated through clonal lineages and that this epigenetic memory can be further modulated by environmental stimuli. To further understand the relationship between the memory and biological functions of AP-1, they constructed transcriptional programs consisting of coregulated genes. Utilizing a clonality measurement approach similar to that used for TFs, they discovered a cell subset with gene expression programs of heritability across entire clones.

The central discovery of this study identifies the AP-1 TF as the primary architect of epigenetic memory. During chronic inflammation, elevated levels of AP-1 proteins like FOS and JUN drive acute tissue repair. Notably, the chromatin accessibility motifs originally opened by AP-1 fail to close even 100 d after inflammatory resolution, despite protein levels returning to baseline across dozens of epithelial turnovers, which is a really surprising revelation. Inhibiting AP-1 activity with the small-molecule inhibitor T-5224 effectively reduced tumor volume in mice recovering from colitis, further confirming the necessity of AP-1 in executing this epigenetic program. Using the deep learning tool seq2PRINT and TF footprinting, they found that AP-1 functions in concert with tissue-specific FOX factors at composite binding sites. AP-1 maintains an open genomic state in stem cells by remodeling chromatin accessibility, which facilitates FOX binding to convert transient inflammatory signals into long-term transcriptional regulatory programs. To determine the targeting process of AP-1, the correlation between gene programs and AP-1 motif accessibility across different clones was further analyzed. These results demonstrated that program 20 (P20) was highly correlated with AP-1 levels, indicating that this epigenetic remodeling centers on the P20 gene program, which includes critical stem cell proliferation genes like *CD44* and *Sox4*. Examination of the P20 gene program within the tissue showed that the expression of AP-1-related genes fell below baseline levels at day 50 and remained suppressed through day 102, with only one statistically different gene persisting after day 50. These findings demonstrate that AP-1 memory is specifically preserved within the epigenome. In these memory cells, the highly accessible chromatin and associated DNA methylation patterns maintain stem cells in a primed state, enabling rapid expansion upon the acquisition of oncogenic mutations.

To evaluate the functional consequences of this memory, Nagaraja et al. induced adenomas via *APC* deletion in mice that had recovered from inflammation. Compared to control groups without a history of inflammation, mice harboring inflammatory memory exhibited markedly accelerated tumorigenesis and increased tumor volumes following the acquisition of *APC* mutations. *APC* mutations effectively triggered the preexisting P20 program, enabling stem cells to facilitate tumor growth by progressively increasing chromatin accessibility to AP-1 and tissue-specific TFs at pro-proliferative gene loci. Overall, this process initiated an accelerated growth phase driven by the synergistic interaction between epigenetic and genetic alterations (Fig. 1).

**Fig. 1.**
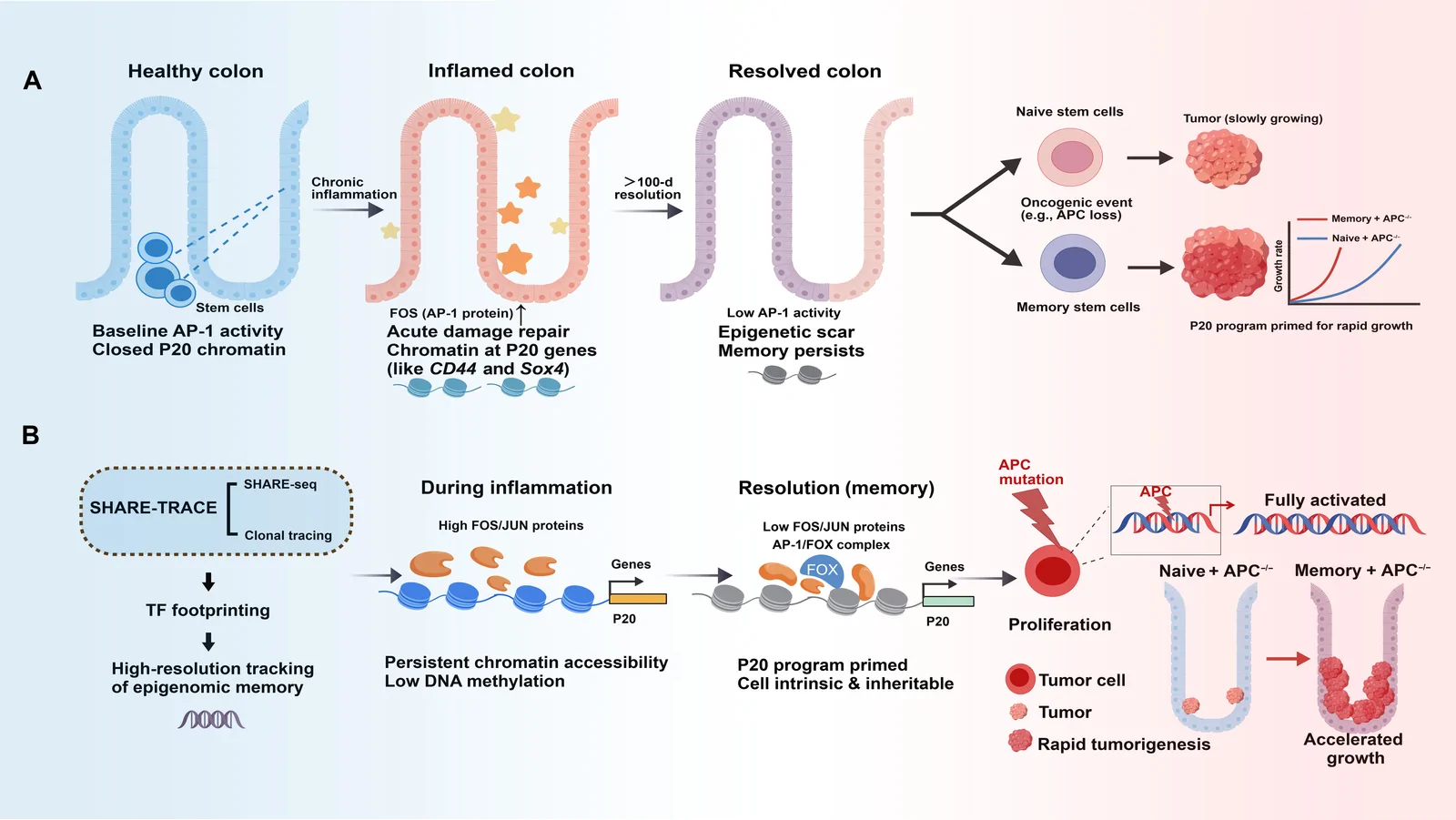
Activator protein 1 (AP-1)-driven epigenetic memory accelerates inflammation-associated tumorigenesis. (A) Overview of inflammatory memory and enhanced tumorigenesis. In the healthy colon, colon stem cells exhibit baseline AP-1 activity, closed chromatin at P20 genes, and high levels of DNA methylation. Chronic inflammation triggers elevated FOS and JUN activity alongside acute damage repair, which leads to the opening of chromatin at P20 genes like *CD44* and *Sox4*. Following long-term resolution exceeding 100 d, the colon regains normal morphology and low AP-1 activity, yet an epigenetic scar persists. Upon an oncogenic event such as the loss of *APC*, these memory stem cells, already primed by the P20 program, exhibit markedly accelerated tumor growth rates compared to naive stem cells. (B) Cell-intrinsic mechanism of epigenetic memory formation and propagation. SHARE-TRACE combines SHARE-seq with cellular barcode-based clonal tracing. Coupled with transcription factor (TF) footprinting, this approach resolves lineage epigenomic memory during colitis-associated tumorigenesis. During inflammation, high levels of FOS and JUN proteins induce the up-regulation of the P20 program, which is characterized by persistent chromatin accessibility and reduced DNA methylation. In the resolution phase, despite low FOS and JUN protein levels, an AP-1/FOX memory complex remains bound to P20 loci, thereby maintaining low DNA methylation and a primed transcriptional state. Subsequent *APC* mutation in these primed cells leads to the robust activation of the P20 program, resulting in tumor-initiating cells with increased proliferation and accelerated tumorigenesis. Created with BioGDP.com and based on concepts from Nagaraja et al. [1].

Collectively, this research establishes “epigenetic inflammation” as a mechanism wherein stem cells retain heritable memory postresolution. Nagaraja et al. elucidated a hierarchical AP-1 and FOX interaction that modulates this memory via chromatin accessibility, creating a primed state that accelerates tumorigenesis upon subsequent oncogenic mutation. While this paradigm deepens our understanding of the inflammation–cancer axis, it necessitates further scrutiny across several critical dimensions. First, the universality of this mechanism across diverse tissue stem cells remains to be validated. A key challenge lies in determining whether this AP-1-dependent memory is a universal response or if distinct inflammatory contexts ranging from acute infections to chronic autoimmune insults leave unique epigenetic “signatures”. Furthermore, while chromatin accessibility and DNA methylation offer initial insights, comprehensive multi-omics integration, including noncoding RNAs and posttranslational modifications, is required to fully resolve the regulatory landscape. Mechanistically, exploring potential links between AP-1-mediated enhancer maintenance and promoter-proximal regulatory events, including plausible impacts on RNA polymerase II pausing, could help bridge the gap between static chromatin snapshots (e.g., ATAC-seq) and dynamic transcriptional activity. Moving beyond deep-learning-based footprinting, future empirical validation through single-cell physical mapping (e.g., single-cell CUT&Tag) will be pivotal to confirm AP-1 occupancy and strengthen causal inferences. Meanwhile, while footprinting provides high-resolution insights, direct validation via targeted chromatin immunoprecipitation sequencing or CUT&RUN in purified memory clones remains the gold standard to eliminate potential computational biases.

Beyond these mechanistic inquiries, it remains to be elucidated how functional heterogeneity within the AP-1 complex, specifically, the distinct roles of various Jun/Fos heterodimers, contributes to the encoding of cellular memory [15]. Equally critical is the potential crosstalk between this AP-1-driven system and canonical pathways such as the nuclear factor-κB axis; determining whether these networks function independently or synergistically will be essential for therapeutic intervention. Finally, assessing the potential for pharmacological “resetting” of these aberrant states, alongside the expansion of current research beyond colorectal models, holds the promise of transforming this framework into a precision medicine strategy. Dissecting the formation, maintenance, and transmission of this memory will ultimately prove decisive in identifying precise therapeutic targets and accelerating the development of clinical strategies for inflammation-associated malignancies.
